# Life after lockdown: Zooming out on perceptions in the post-videoconferencing era^☆^^☆☆^^★^

**DOI:** 10.1016/j.ijwd.2021.08.009

**Published:** 2021-08-27

**Authors:** Channi Silence, Shauna M. Rice, Samara Pollock, Janet E. Lubov, Linda O. Oyesiku, Sonya Ganeshram, Alexa Mendez, Freyja Feeney, Arianne Shadi Kourosh

**Affiliations:** a Massachusetts General Hospital, Department of Dermatology, Boston, Massachusetts; b University of Massachusetts Medical School, Worcester, Massachusetts; c University of Maryland, Department of Medicine, Baltimore, Maryland; d Wright State University, Boonshoft School of Medicine, Dayton, Ohio; e Holy Cross Hospital, Fort Lauderdale, Florida; f Harvard University, Boston, Massachusetts; g Herricks High School, Long Island, New York; h University of Vermont, Burlington, Vermont; i Harvard Medical School, Boston, Massachusetts

**Keywords:** Esthetics, Body dysmorphia, Cosmetic dermatology, Self-perception

## Abstract

**Background:**

The COVID-19 pandemic has deeply disrupted daily life across the globe, with profound effects on mental and physical health. After more than a year of isolation and communication via videoconferencing, people are returning to in-person activities.

**Objective:**

This study aimed to investigate worsening self-perception, mental health, and anxiety with the return to in-person activities, with a focus on the influence of videoconferencing, social media, and the use of filters.

**Methods:**

An anonymous survey was distributed online through social media platforms and student network pages.

**Results:**

A total of 7295 participants responded to the survey. Seventy-one precent reported anxiety or stress related to returning to in-person activities, and nearly 64% sought mental health support services. Thirty-percent stated they plan to invest in their appearance as a coping strategy to deal with the anxiety of returning to in-person, and >30% plan to take action in changing their appearance. The most reported dermatologic concerns were skin discoloration (32.36%), wrinkles (24.45%), and acne (14.85%). The prevalence of anxiety and mental health services increased relative to the use of filters in 18- to 24 year-olds.

**Conclusion:**

This survey study of >7000 participants across the country elucidates worsening self-perception, anxiety, and mental health as we return to in-person activities in relation to increased videoconferencing, social media usage, and the use of filters. Physicians should be aware of these effects to better serve their patients.

**What is known about this subject in regard to women and their families?**

• Body dysmorphic disorder in women has been on the rise during the pandemic and worsened with increased use of videoconferencing.
• Videoconferencing has been cited as a cause of the increase in cosmetic consultations among women during the pandemic.
• Very few studies have addressed the increase in videoconferencing during the COVID-19 pandemic and the resulting effects on mental health and self-perception.

**What is new from this article as messages for women and their families?**

• Increased time spent videoconferencing, using social media, and using filters on these platforms during the pandemic has led to worsening self-perception and mental health, especially in younger girls and women.
• Families should be aware that increased social media and filter use may lead to elevated levels of anxiety in teens and college-age children, especially as activities resume in-person for the first time in a year and a half.
• Women may be more apt to change their appearance as a method of coping with the anxiety associated with resuming in-person activities after a year spent communicating virtually.

Alt-text: Unlabelled box

## Introduction

After a year and a half of living through the COVID-19 pandemic, lockdowns and social distancing, remote school and work, and life spent on videoconferencing, for many the long-awaited return to in-person activities is finally within sight. Reflecting on this return also inspires reflection on how the changes in the world and in our daily lives during this time have affected both individuals and society in ways that may be enduring.

In 2020, as the outbreak of COVID-19 rapidly shifted life and work virtually, >3 trillion hours were spent on Zoom (Dean, 2021). With this, we saw increased scrutiny of personal perception as people viewed themselves for hours on front-facing cameras that distort appearance in ways often unknown to viewers, leading many to seek cosmetic procedures they had not considered before (Rice et al., 2021). Meanwhile, the National Eating Disorders Association reported increases as high as 70% to 80% in calls to its helpline (Katella, 2021), with concerns that the increased use of videoconferencing technologies indirectly contributed to the risk of eating disorders by increasing preoccupation with appearance (Rodgers et al., 2020). Numerous reports indicated that patients with anorexia, binge-eating disorders, and substance use disorders experienced worsening symptoms throughout the pandemic (Schlegl et al., 2020), and Google searches for hair loss treatment, including minoxidil and platelet-rich plasma, reached all-time highs. Minoxidil was searched >40% more than prior years (Gupta, et al., 2021). A health poll from July 2020 found that many adults had difficulty sleeping (36%) or eating (32%) and experienced worsening of chronic conditions (12%; Kaiser Family Foundation, 2021). Substance use, especially alcohol, surged during the pandemic, as evidenced by online alcohol sales increasing by 262% (Pollard, 2020). The Overdose Mapping and Application Program, a reporting system for overdose detection, reported an 18% increase in the number of overdoses during the early months of the pandemic compared with the same time period the year prior (American Psychological Association, 2021). Many of these negative effects were triggered by a lack of structure and of social support throughout lockdown and exacerbated by the fact that videoconferencing heightened focus on appearance.

Today, we find ourselves at the next phase, with loosening of COVID-19 restrictions and the return to in-person activities. The impacts of the pandemic on social, mental, and physical health are now being viewed in the eye of the public, building on preexisting anxieties. In this study, we investigate the effects that virtual life has caused on self-perception and mental health as we return to in-person socialization.

## Methods

In this institutional review board–approved study, a voluntary anonymous survey was made available online to participants throughout the United States. Participants were queried about changes in self-perception, mental health, and anxiety upon return to in-person activities, with a focus on the influence of videoconferencing, social media, and the use of filters. Survey development began with qualitative interviews between college- and graduate school–age authors and their peers to identify relevant questions. The survey was reviewed for content and face validity by a statistician and was made available via the social media platforms of all authors and posted on various student network pages. Study data were collected and managed using REDCap electronic data capture tools hosted by Lifespan's Department of Information Services.

## Results

**Fig. 1:**
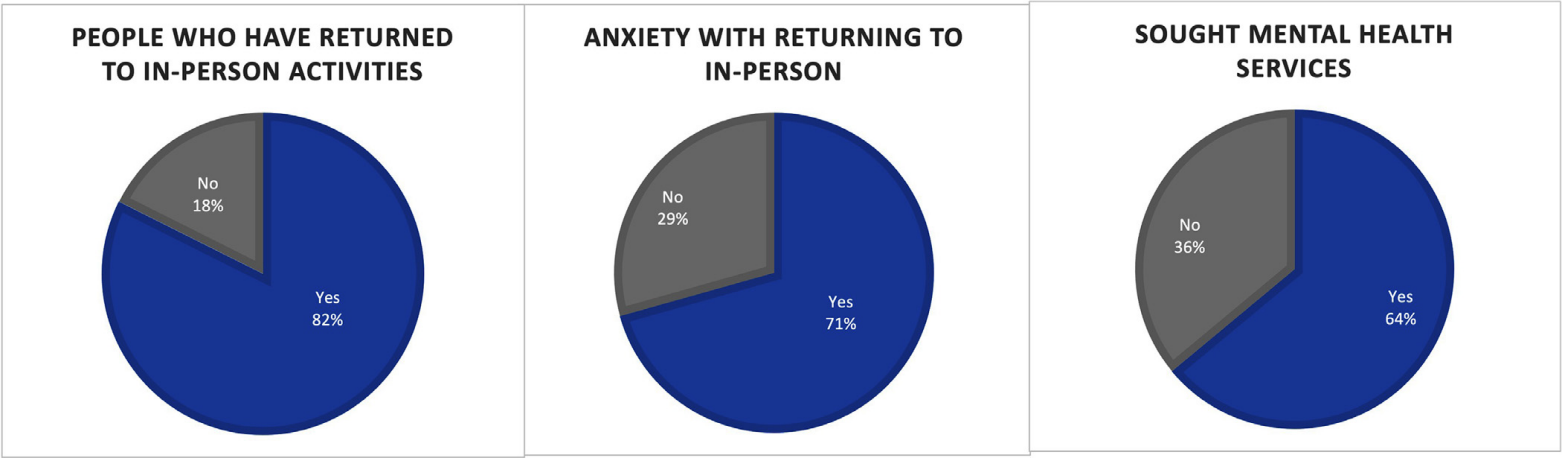
Anxiety in returning to in-person activities and seeking mental health services among all respondents.

**Fig. 2:**
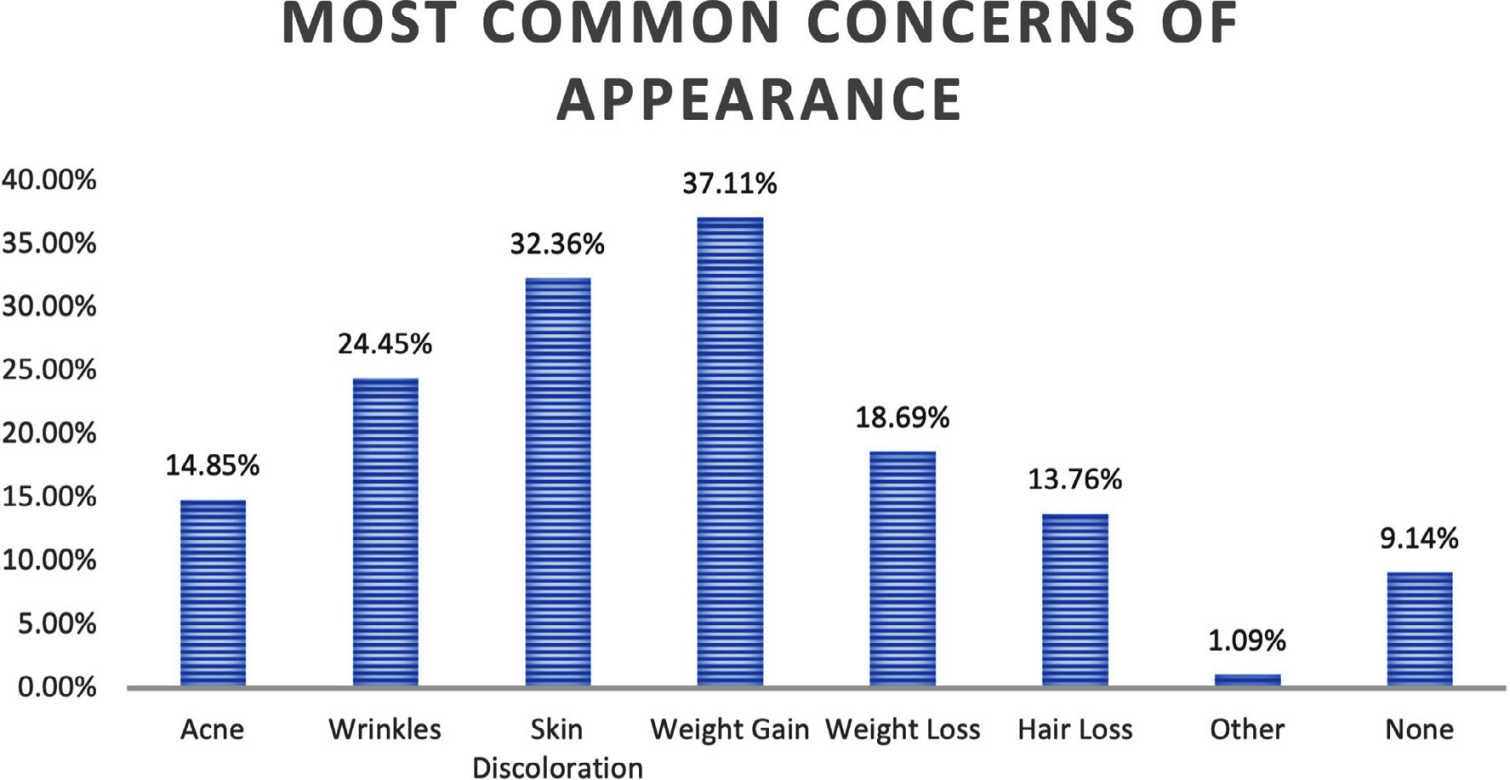
Most common concerns of appearance in resuming in-person activities among all respondents.

**Fig. 3:**
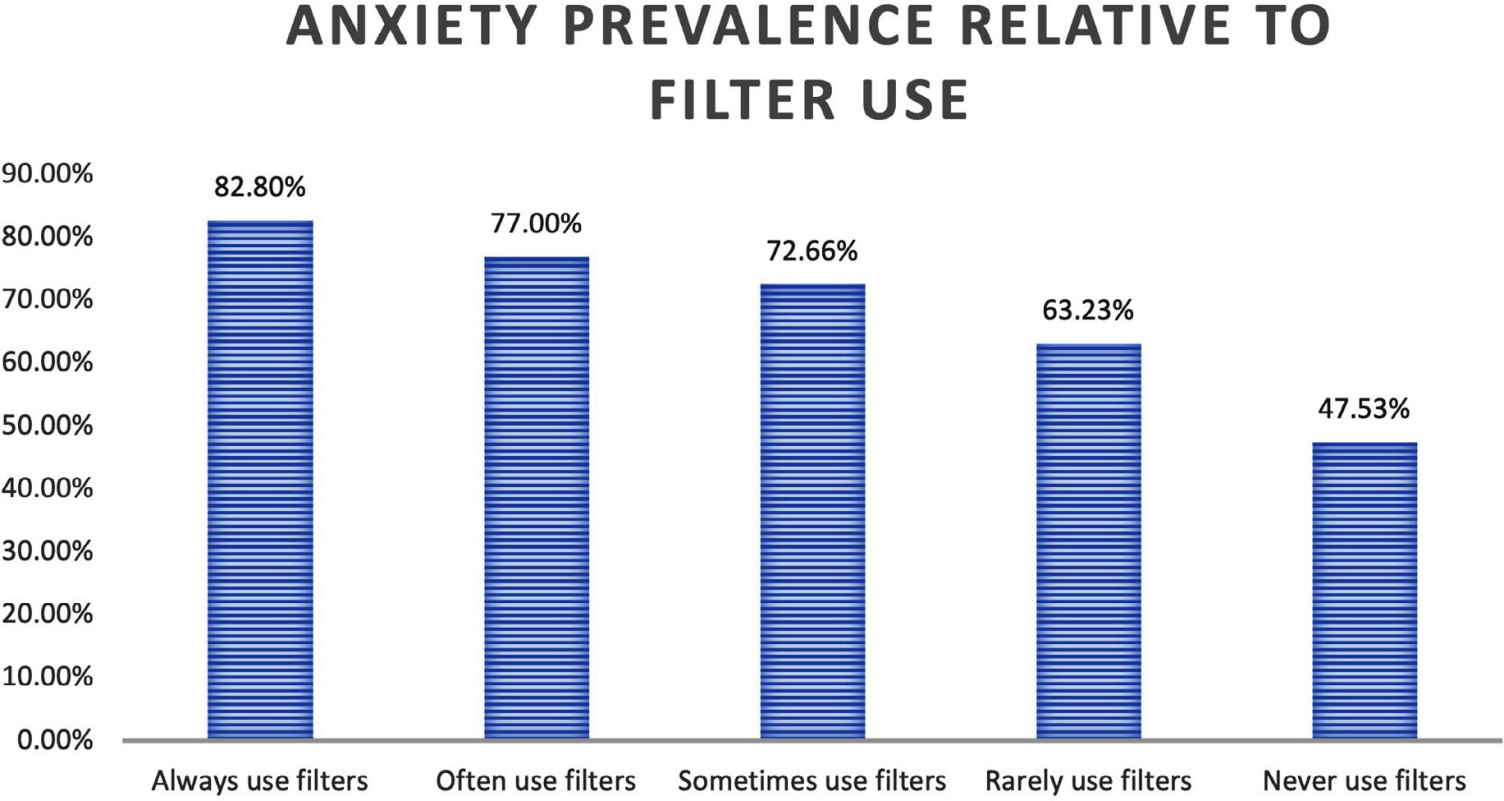
Prevalence of anxiety relative to frequency of filter use in all respondents.

A total of 7295 participants responded to the survey. Demographic information is listed in Table 1. Of the participants, 82.4% reported returning to in-person activities, with 70.6% reporting anxiety or stress with this return, and nearly 64% sought mental health support services (Fig. 1). Nearly 30% of all participants stated that they planned to invest in their appearance as a coping strategy to deal with the anxiety of returning to in-person activities, and >30% planned to take action in changing their appearance. The most common physical concerns regarding appearance included weight gain (37.1%), skin discoloration/scarring (32.36%), and wrinkles (24.5%; Fig. 2). The prevalence of anxiety increased relative to the increased use of filters on social media (Fig. 3).

**Table 1:** Demographic data

	Variable	n (%)
Age, y	18–24	1294 (17.8)
	25–34	3941 (54.1)
	35–44	1505 (20.67)
	45–54	412 (5.7)
	55–64	95 (1.3)
	65–74	29 (0.4)
	≥75	5 (0.1)
Gender		
	Male	3593 (49.6)
	Female	3208 (44.3)
	Nonbinary	355 (4.9)
	Transgender	83 (1.1)
	Other	6 (0.1)
Ethnicity		
	American Indian or Native American	679 (9.4)
	Asian	811 (11.3)
	Black or African American	1160 (16.1)
	Native Hawaiian or Pacific Islander	582 (8.1)
	White	3845 (53.4)
	Hispanic or Latino	725 (10.1)
	Other	42 (0.6)
Education/professional status		
	High school	395 (5.5)
	College	3060 (42.3)
	Graduate school	2850 (39.4)
	Workforce	893 (12.4)
	Other	29 (0.4)
Region		
	Midwest	1183 (16.4)
	Northeast	2286 (31.6)
	Southeast	2097 (29.0)
	Southwest	821 (11.4)
	West	837 (11.6)

**Fig. 4:**
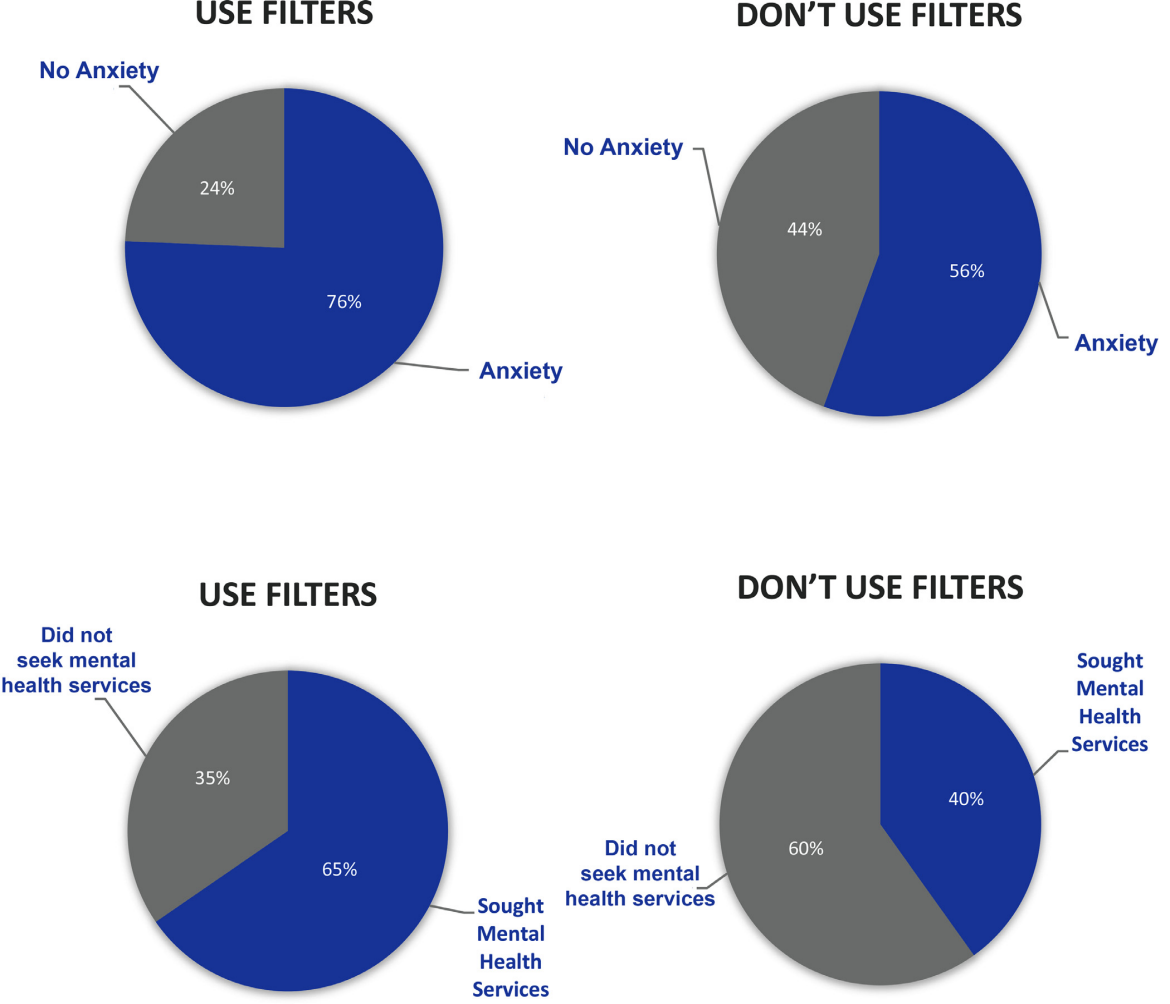
Anxiety and seeking mental health services in respondents age 18 to 24 years who use filters compared with those who do not.

**Fig. 5:**
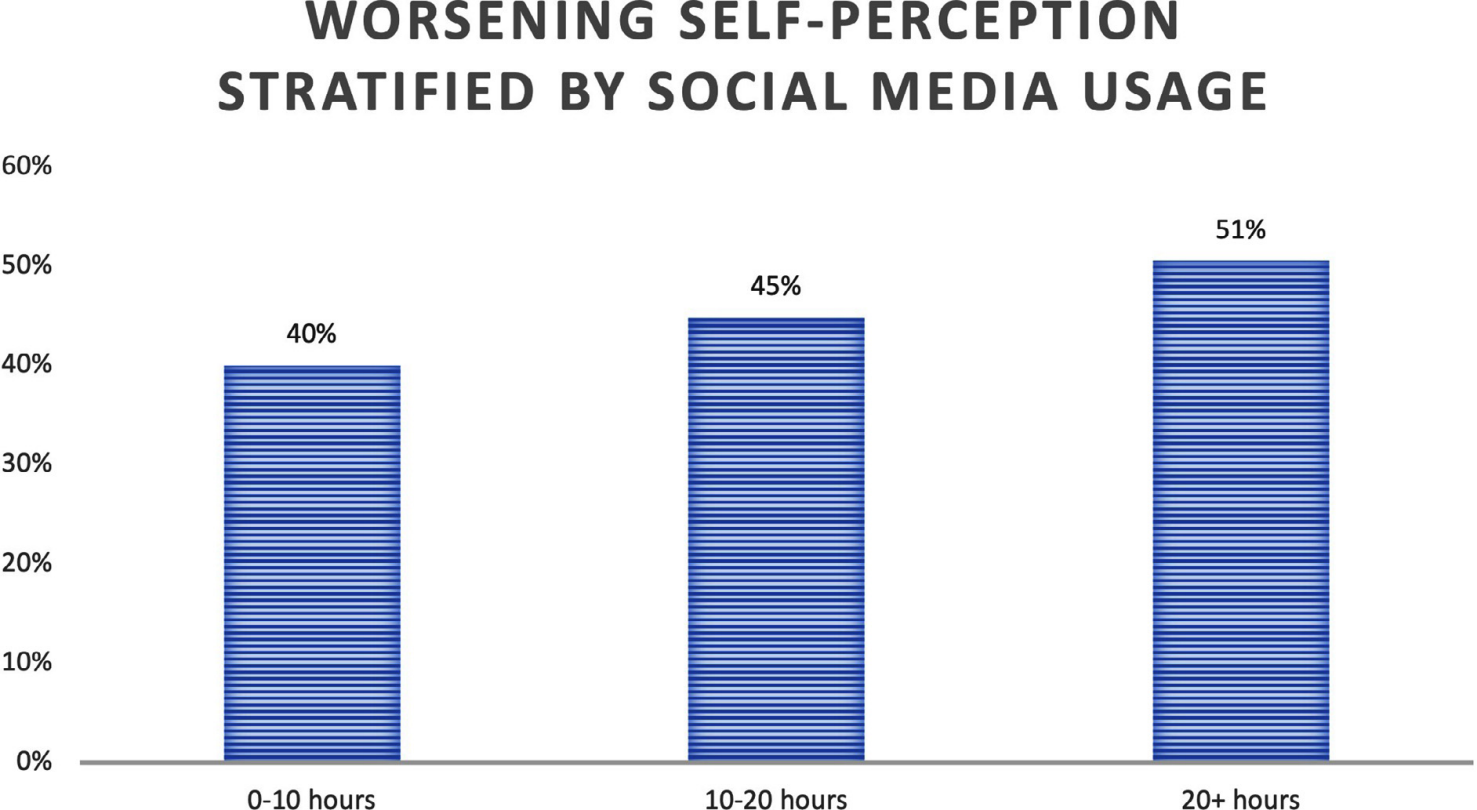
Worsening self-perception in respondents age 18 to 24 years, stratified by hours of social media usage per week.

Among the participants, 1294 (17.77%) were age 18 to 24 years. In this age group, 45% felt worse about their appearance. Higher levels of anxiety and mental health services were noted in individuals age 18 to 24 years who felt worse about their appearance and those who used filters on social media (Fig. 4). Forty-one percent of this college-aged cohort reported apprehension regarding social interactions with the return of in-person activities, and 39.1% reported feeling self-conscious about their appearance. Increased time spent on social media and videoconferencing in 18- to 24 year-olds resulted in worsening anxiety and increased use of mental health services. Among this age group, as time spent on social media increased, so did worsening self-scrutiny. Forty percent of 18- to 24-year-olds who spent 0 to 10 hours on social media, 45% who spent 10 to 20 hours, and 51% who spent >20 hours endorsed worsening self-perception (Fig. 5). Among 18- to 24-year-olds who used filters while videoconferencing, higher levels of anxiety were reported about returning to in-person activities (85% vs. 58% in nonfilter users). Thirty-seven percent of people age 18 to 24 years stated that they planned to invest in their appearance as a coping strategy to deal with the anxiety of returning to in-person activities, and 38% planned to take action in changing their appearance, which is higher than the general population surveyed in this study. Filters on social media (45.6%), improving appearance for in-person activities (45.6%), the appearance of others in real life (45.1%), and others on social media (40.5%) were listed as major contributing factors to change appearance in participants age 18 to 24 years.

## Discussion

The shockwave effects of the COVID-19 pandemic across mental, emotional, and physical health (Holmes et al., 2020; Rogers et al., 2020) are only beginning to be understood. The stress faced during the pandemic has played out in a variety of ways, with increases in calls to eating disorder and mental health hotlines (Katella, 2021), surges in substance use (Schlegl et al., 2020) and depression (Kaiser Family Foundation, 2021), and even an increase in cosmetic consultations due to heightened concerns regarding appearance on screen (Rice et al., 2021). Research on the health effects of the COVID-19 pandemic is paramount and prompted us to explore how these recent life events and changes have influenced self-perception and mental health as face-to-face life returns (Holmes et al., 2020).

In this survey study of >7000 individuals across the country, nearly 70% of respondents indicated stress or anxiety related to the return to in-person activities, with concerns of appearance as a significant source of the anxiety. Our results show that certain behaviors and activities used during the pandemic may make some individuals more susceptible to such stress, anxiety, and concerns of self-perception. For example, those who used filters on social media or videoconferencing calls during the pandemic tended to feel worse about their appearance and had overall higher levels of anxiety with the return of in-person activities. In this survey, >70% of people used filters on social media and videoconferencing, highlighting the desire to have an edited appearance, alter skin texture and face contour, and eliminate signs of aging to maintain esthetic features.

The subset of participants age 18 to 24 years appears to be the most influenced by this trend, with 64% of those who used filters reporting concerns regarding appearance versus only 37% of those who did not use filters. Furthermore, young adults who used filters reported higher levels of anxiety (85%) compared with their counterparts (58%). There is a higher level of dependence for young adults to use photo-altering features in these apps to enhance their appearance (American Psychological Association, 2020), and when learning and socializing was conducted via videoconferencing during the pandemic, the use of filters became even more the norm.

At a time when technology has been essential to prevent the spread of the virus, it also has its drawbacks. Social media use appears to influence anxiety and self-perception, with those spending >20 hours per week on social media reporting higher levels of anxiety as the world reopens compared with those spending less time on social media platforms. This aligns with findings of previous studies showing elevated body dissatisfaction associated with increased engagement on social media (Shome et al., 2020). In a study by the American Psychological Association (2020), young adults experienced elevated levels of stress (43%) and feelings of loneliness (63%) during the pandemic, although they were the “most connected” of any generation. Younger individuals spend more time on social media, and hours spent online have increased to an average of 2.5 hours per day (Henderson, 2021). TikTok gained 85% of its followers during the pandemic (Business of Apps, 2021), and Snapchat reported that 200 million of its users apply a filtered lens daily (Aslam, 2019). The continuous flow of altered images and high expectations of appearance cause further anxiety and mental health issues within our communities.

Study participants age 18 to 24 years were surveyed regarding their use of mental health services throughout the pandemic, and interestingly, 65% of those who reported using filters sought mental health services compared with 40% for those who did not (Fig. 4). Almost half of participants age 18 to 24 years surveyed felt self-conscious about their appearance, and those who felt negatively about their appearance also had higher levels of anxiety (82%). At a time when the rate of mental health conditions is increasing, with approximately 30% of individuals age 18 to 25 years having a mental illness (U.S. Department of Health and Human Services, 2021), it is pertinent that we pay attention to risks and causes of negative feelings. Our data demonstrate that increased social media hours and the use of filters are correlated with negative self-perception, anxiety, and an increased need for mental health services. However, during the COVID-19 pandemic, students were expected to attend college virtually and learn through online platforms to an unprecedented degree, possibly worsening their self-image and mental health.

In our study, the most frequently reported dermatologic concerns were skin discoloration (32.36%), wrinkles (24.45%), and acne (14.85%). The use of videoconferencing added a level of scrutiny to self-image as people watched their own reflection for hours per day. Continuous visual feedback from the self-pointing camera highlightss facial attributes that would not have been analyzed previously, enhancing the appearance of skin laxity, discoloration, and facial asymmetry (Třebický et al., 2016). Thirty-seven percent of respondents in this survey also noted concerns of weight gain. A study conducted in the United Kingdom demonstrated that the pandemic resulted in increased maladaptive eating behaviors, body dissatisfaction, and restrictions on exercise, leading to weight gain and body shame (Robertson, 2021).

Although measures were taken to reduce bias and gather responses from a wide range of people, there are possible limitations to this study. Surveys were distributed through social media platforms, possibly skewing the population toward individuals who spend more time on social media and those who are more engaged with online content.

As we return to in-person social and professional activities, it is important to consider the effects of the pandemic on self-esteem and visual appearance. In particular, changes in self-perception and anxiety as a result of constant videoconferencing may lead to unnecessary cosmetic procedures, especially in young adults who have had increased exposure to online platforms, including videoconferencing, social media, and filters, throughout the pandemic. In our survey, approximately 37% of respondents age 18 to 24 years stated that they planned to invest in their appearance as a coping strategy for anxiety, and 38% planned to take action in changing their appearance. Dermatologists and the general medical community should recognize these possible motivations in patients seeking cosmetic treatments. However, in the midst of Zoom dysmorphia, it was affirming to see how our community of cosmetic physicians approach concerns of self-image, with the caring eye of doctors as stewards of esthetic medicine.

Now, as we move beyond lockdown, it is evident that prolonged time on videoconferencing and social media, as well as the use of filters on these platforms, has affected feelings of anxiety, self-consciousness, and mental health as life resumes in person.

## Conclusion

During the COVID-19 pandemic, videoconferencing and social media became the primary means of communication to connect with friends and coworkers. This transition to a digital world led to the unintended effect of increased hours of self-scrutiny as people stared at their own reflections and compared themselves with others on the screen. In this survey study of >7000 participants, increased hours spent on videoconferencing and use of filters elucidated worsening anxiety, mental health, and self-perception as we return to in-person activities. As we re-enter a life of socializing, esthetic physicians and the medical community at large should be aware of the effects of increased videoconferencing related to worsening mental health and self-perceptions to better serve our patients.
